# Causes and risk factors for infant mortality in Nunavut, Canada 1999–2011

**DOI:** 10.1186/1471-2431-12-190

**Published:** 2012-12-12

**Authors:** Sorcha A Collins, Padma Surmala, Geraldine Osborne, Cheryl Greenberg, Laakkuluk Williamson Bathory, Sharon Edmunds-Potvin, Laura Arbour

**Affiliations:** 1 Department of Medical Genetics, University of British Columbia Island Medical Program, University of Victoria, PO Box 1700 STN CSC, Victoria, BC, V8W 2Y2, Canada; 2 Court Services Division, Department of Justice, Government of Nunavut, Iqaluit, NU, Canada; 3 Department of Health and Social Services, Government of Nunavut, Iqaluit, NU, Canada; 4 Department of Pediatrics and Child Health, University of Manitoba, Winnipeg, MB, Canada; 5 Nunavut Tunngavik Incorporated, Iqaluit, NU, Canada

**Keywords:** Inuit, Nunavut, Aboriginal, Infant mortality, Sudden infant death syndrome, Sudden unexpected death in infancy, Carnitine palmitoyltransferase 1 deficiency, CPT1A P479L variant

## Abstract

**Background:**

The northern territory Nunavut has Canada’s largest jurisdictional land mass with 33,322 inhabitants, of which 85% self-identify as Inuit. Nunavut has rates of infant mortality, postneonatal mortality and hospitalisation of infants for respiratory infections that greatly exceed those for the rest of Canada. The infant mortality rate in Nunavut is 3 times the national average, and twice that of the neighbouring territory, the Northwest Territories. Nunavut has the largest Inuit population in Canada, a population which has been identified as having high rates of Sudden Infant Death Syndrome (SIDS) and infant deaths due to infections.

**Methods:**

To determine the causes and potential risk factors of infant mortality in Nunavut, we reviewed all infant deaths (<1yr) documented by the Nunavut Chief Coroner’s Office and the Nunavut Bureau of Statistics (n=117; 1999–2011). Rates were compared to published data for Canada.

**Results:**

Sudden death in infancy (SIDS/SUDI; 48%) and infection (21%) were the leading causes of infant death, with rates significantly higher than for Canada (2003–2007). Of SIDS/SUDI cases with information on sleep position (n=42) and bed-sharing (n=47), 29 (69%) were sleeping non-supine and 33 (70%) were bed-sharing. Of those bed-sharing, 23 (70%) had two or more additional risk factors present, usually non-supine sleep position. CPT1A P479L homozygosity, which has been previously associated with infant mortality in Alaska Native and British Columbia First Nations populations, was associated with unexpected infant death (SIDS/SUDI, infection) throughout Nunavut (OR:3.43, 95% CI:1.30-11.47).

**Conclusion:**

Unexpected infant deaths comprise the majority of infant deaths in Nunavut. Although the CPT1A P479L variant was associated with unexpected infant death in Nunavut as a whole, the association was less apparent when population stratification was considered. Strategies to promote safe sleep practices and further understand other potential risk factors for infant mortality (P479L variant, respiratory illness) are underway with local partners.

## Background

Nunavut, a northern Canadian territory covering the most northern and eastern area of Canada, is Canada’s largest jurisdictional land mass with 33,322 inhabitants, of which 85% self-identify as Inuit [1,2]. Nunavut leads the country in adverse early child health outcomes such as infant mortality [3], congenital anomalies [4], prematurity, and low birth weight [5,6]. The infant mortality rate in the territory (14.6/1,000 live births) is three times higher than the national average (5.2/1,000 live births) and twice that of the Northwest Territories (1999–2009) [3].

An estimated 850 infants are born in Nunavut each year, with 90-95% of those being born to Inuit women [2,5]. There are 25 communities in the three regions of Nunavut: Qikiqtani, Kivalliq and Kitikmeot. Most Nunavut communities are isolated geographically and are accessible only by air and sea. Acute medical care is delivered out of territory in Ontario (Qikiqtani), Manitoba (Kivalliq), and the Northwest Territories (Kitikmeot) [7,8]. Approximately 45% of births to Nunavut residents occur out of territory [9].

In 2010, Luo et al. [10] reported that Sudden Infant Death Syndrome (SIDS) and infection were the leading causes of infant death in Inuit-inhabited regions of Canada between 1990 and 2000. SIDS is a diagnosis of exclusion where the cause of death remains unexplained after a thorough investigation, including a complete autopsy, examination of the death scene, and review of clinical history [11]. Sudden Unexpected Death in Infancy (SUDI) is a broader category that includes unexpected infants deaths with other risk factors present, such as an illness or risk factors for asphyxia [12,13].

The causes of SIDS and SUDI are multi-factorial, due to a combination of environmental, developmental and genetic factors [12-15]. Reducing the risk from one factor may decrease the overall risk of SIDS. Placing infants to sleep on their backs (supine) has substantially reduced SIDS worldwide, apparently over-riding inherent risk factors and reducing risk of asphyxia [13,14]. Results from surveys of Nunavut mothers suggest that only 38-46% of mothers place their infants to sleep on their backs, compared to 77% for the rest of Canada [16,17]. While it is controversial whether bed-sharing (sharing a sleep surface with an infant) is a risk factor itself, bed-sharing is associated with increased risk for SIDS when combined with other risk factors, including non-supine sleep position, bed-sharing with a non-caregiver and bed-sharing with a parent/caregiver who smokes or has impaired arousal [18,19].

Undiagnosed fatty acid oxidation disorders account for 3-6% of SIDS and SUDI cases [15,20,21]. The P479L (c.1436C>T) genetic variant of the hepatic fatty acid oxidation enzyme, carnitine palmitoyltransferase 1A (CPT1A), has been associated with infant mortality in Alaska Native and British Columbia coastal First Nations populations, where the variant is common [22,23]. In 2010, we established that the CPT1A P479L variant is highly prevalent in Nunavut; more than 70% of infants born in Kitikmeot and Kivalliq regions of Nunavut were P479L homozygous (n=290; 2006) [24]. To date, the P479L variant has only been reported in Indigenous populations [24,25]. Importantly, the variant has also been reported to confer positive effects in studies of cardiovascular lipid profiles and obesity [25,26]. It remains controversial whether the variant is a risk factor itself for infant mortality or a marker of other population-based risk factors more common in high prevalence areas.

Reducing infant mortality is a primary goal of the 2008–2013 Government of Nunavut Public Health Strategy, *Developing Healthy Communities*[27]. In keeping with the goal of developing programs to reduce infant mortality in Nunavut, we report the causes and associated risk factors in Nunavut from 1999–2011, using data from the Nunavut Chief Coroner’s Office, Nunavut Bureau of Statistics and Statistics Canada [28]. We also compare Nunavut rates to published national data for similar years available through the Public Health Agency of Canada [29].

## Methods

### Ethics

Ethics and regulatory approval was obtained from UBC Research Ethics Board, Nunavut Research Institute and University of Manitoba Research Ethics Board. The review was conducted in partnership with Nunavut Tunngavik Inc.

### Data sources

All infant deaths (live birth with death before 1 year of age) occurring in Nunavut between July 1, 1999 and June 30, 2011 and reported to the Nunavut Chief Coroner’s Office and, subsequently, to the Nunavut Chief Medical Officer of Health, were reviewed. Infant deaths recorded by the Nunavut Bureau of Statistics were also reviewed. This dataset captured all postneonatal infant death and a subset of neonatal death. Early neonatal deaths for those infants born out of territory where death occurred before registration in Nunavut were not commonly available. Consequently, ascertainment of perinatal and neonatal deaths was limited for this review. All prenatally occurring deaths (stillbirths) were excluded from this review.

Variables collected (when available) were: date of birth, date of death, cause of death, gender, gestational age at birth, mother’s place of residence at time of death, CPT1A P479L genotype, and sleep environment (i.e. sleep position, sleep surface, loose bedding, bed-sharing). Sleep environment was obtained from death scene investigation report. Bed-sharing was defined as those infants sharing a sleep surface with another person as documented when the death occurred. Causes of death were as determined by the Chief Coroner after review of autopsy reports and death scene investigation. Cases were grouped into one of four major cause of death categories; SIDS/SUDI, infection (respiratory, H influenza or other), congenital anomaly or other.

Data for live births to mothers residing in Nunavut for July 1, 1999-June 30, 2011 (n=9215) were obtained from Statistics Canada [28]. National comparison data for infant deaths (1998–2007) were obtained from the Public Health Agency of Canada 2011 Perinatal Health Indicators for Canada report [29].

When available, CPT1A genotype data for the P479L variant was collected for unexpected infant death cases (SIDS/SUDI and death due to infection) where sub-optimal CPT1A function might influence outcome. There were 81 unexpected infant death cases during the review period and CPT1A P479L genotype information was available for 35 cases. In 2004, the Manitoba Newborn Screening program commenced a pilot CPT1A P479L newborn screening program for infants born to Kivalliq region residents [30]. Eighteen of the 35 identified cases had been previously genotyped during routine newborn screening as part of that pilot project. The remaining 17 were genotyped on request of the Chief Coroner or pathologist performing the autopsy. Our previously reported data for CPT1A P479L genotypes in infants born to Nunavut residents in 2006 (n=695) were used for comparison [24].

### Data analysis

Crude mortality rates were calculated per 1,000 live births by region and for Nunavut [28,31]. Nunavut rates were compared to Canadian data using odds ratios with 95% confidence intervals (CI). Crude national infant and postneonatal mortality rates were calculated with available 1998–2007 data, and crude national cause-specific mortality rates were calculated with available 2003–2007 data. National rates exclude Ontario [29].

Association (odds ratios with 95% CI) of the P479L variant with unexpected infant death (SIDS, SUDI and infection) was analysed by comparing P479L homozygous cases to the population P479L homozygosity, as estimated for live born infants undergoing newborn screening in Nunavut in 2006 [24]. Population data were analysed by comparing P479L homozygosity between regions using χ^2^ test with a *p*<0.05 significance level. Hardy-Weinberg equilibrium was previously calculated [24].

All statistical analysis was conducted using STATA 11 (StataCorp. 2009. *Stata Statistical Software*: *Release 11*. College Station, TX). Two-tailed *p* values <0.05 were considered significant. Due to limited information, risk associated with sleep practices could not be analysed. Information on maternal and household characteristics, including parity, maternal age, maternal education, household income and food security, were very limited or not available for this review.

## Results

There were 117 infant mortality cases documented in Nunavut between 1999 and 2011. Ninety infant deaths occurred in Nunavut and were reported to the Chief Coroner. An additional 27 cases were reported to the Nunavut Bureau of Statistics, 7 of which occurred in the territory during the perinatal period (all occurred on the first day of life); the remaining 20 occurred out of territory (Edmonton, Winnipeg and Ottawa). Cause of death was documented for 95 cases.

The leading causes of infant death were SUDI (n=32; 27%), SIDS (n=24; 21%) and infection (n=25; 21%). Combined, SIDS and SUDI (SIDS/SUDI) comprised the majority of cases (48%). Cause-specific infant mortality rates for SIDS/SUDI and infections were 6.08 and 2.71/1,000 live births, respectively, and these rates were significantly increased compared to national rates (ORs:11.97; 95%CI: 8.9-15.8 and 8.79; 95%CI: 5.6-13.2; Table 1). Cause specific mortality rates for SIDS/SUDI and respiratory infections were higher in the Kitikmeot region than in the Kivalliq and Qikiqtani regions.

**Table 1:** **Age and cause specific mortality rates** (**per 1**,**000 live births**) **for infant deaths documented in Nunavut by region** (**n**=**117**; **July 1 1999**-**June 30 2011**) **and in Canada**^†^ (**1998**–**2007**)

	Nunavut IMR (95% CI)*				**Canada**^**†**^**IMR (95% CI)**	**Odds Ratio (95% CI)**
	**Qikiqtani**	**Kivalliq**	**Kitikmeot**	**Nunavut**		
Live births	4,859	2,817	1,539	9,215	1,065,647	
**Mortality Rates**						
Infant death	10.91 (8.18-14.24)	13.13 (9.26-18.06)	16.89 (11.06-24.66)	12.70 (10.51-15.20)	5.15 (5.01-5.28)	2.47 (2.04-2.98)
Postneonatal death	8.23 (5.89-11.19)	10.65 (7.20-15.17)	11.70 (6.95-18.42)	9.66 (7.76-11.87)	1.39 (1.31-1.46)	6.96 (5.56-8.64)
**Cause of Death**						
SIDS and SUDI	5.76 (3.83-8.32)	5.68 (3.25-9.21)	7.80 (4.04-13.58)	6.08 (4.59-7.88)	0.51 (0.47-0.55)	11.97 (8.92-15.79)
Infection	2.06 (0.99-3.78)	3.19 (1.46-6.06)	3.25 (1.06-7.57)	2.71 (1.76-4.00)	0.31 (0.28-0.34)	8.79 (5.60-13.21)
Respiratory	0.82 (0.22-2.11)	0.71 (0.09-2.56)	1.95 (0.40-5.69)	1.63 (0.91-2.68)	-	-
H influenza	0.41 (0.05-1.49)	0.36 (0.01-1.98)	1.30 (0.16-4.69)	0.65 (0.24-1.42)	-	-
Other	0.82 (0.22-2.11)	0.00 (0.00-1.31)	0.00 (0.00-2.39)	0.43 (0.12-1.11)	-	-
Congenital anomalies	0.41 (0.05-1.49)	0.36 (0.01-1.98)	1.30 (0.16-4.69)	0.54 (0.18-1.27)	1.19 (1.13-1.26)	0.46 (0.15-1.07)

* Crude mortality rates for Nunavut were calculated per 1,000 live births.

^†^ Canadian rates (excluding Ontario) of crude infant and postneonatal mortality were calculated with available 1998–2007 data and cause-specific mortality were calculated with available 2003–2007 data, as reported by the Public Health Agency of Canada in the Perinatal Health Indicators for Canada 2011 report [29].

*IMR* = infant mortality rate. *CI* = confidence interval.

**Figure 1:**
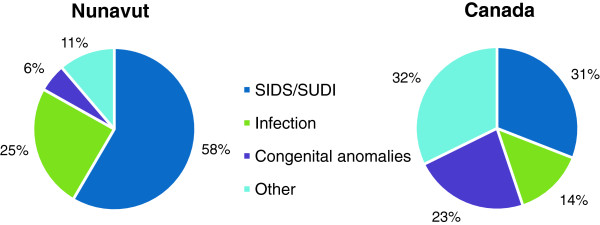
**Causes of Postneonatal Death in Nunavut(July 1 1999-June 30 2011; n=89)and Canada(excludes Ontario; 2003–2007; n=1478) [**[29]**].** Infection includes deaths due to respiratory or other infections.

Postneonatal deaths (28–364 days) accounted for 76% (n=89) of all infant deaths in this review. The crude postneonatal mortality rate for Nunavut was 9.66/1,000 live births, significantly higher than the national rate of 1.39 (OR=6.96, 95%CI: 5.56-8.64). SIDS/SUDI (n=52, 58%) and infection (n=22, 25%) were the leading causes of postneonatal death in Nunavut (Figure 1).

### SIDS and SUDI risk factors

There were 56 SIDS and SUDI cases during the review period; 86% occurred before 6 months of age. Deaths were evenly distributed between genders (male=29, female=27). Gestational age at birth was available for 41 cases, 14 (25%) were born premature (<37 completed weeks of gestation).

Some information on sleep environment at time of death was available for 52 of the 56 SIDS/SUDI cases. Two or more sleep environment risk factors were documented in 39 cases (70%). Of those cases with documented sleep position at time of death (n=42), sleep position was non-supine for 29 (69%). Bed-sharing occurred in 33 of the 47 cases (70%) with that information. Of the 33 cases known to be bed-sharing at time of death, 23 (70%) had two or more additional risk factors present, including non-supine sleep position, sleeping on a soft surface (sofa), bed-sharing with a non-caregiver, and/or smoking or alcohol/illicit drug use by bed-sharer.

### Death due to infection

Death due to infection was the cause of death for 25 cases. The majority of deaths were due to respiratory infection (n=15) or *Haemophilus influenzae* infection (type a or b; n=6). Two *Haemophilus influenzae* cases had a respiratory component. The remaining infectious causes included herpes simplex virus, granulomatous encephalitis, sepsis and viral myocarditis.

### Prematurity

Gestational age was available for 75 of the total infant mortality cases, of those, 29 (39%) were born premature (<37 completed weeks of gestation), representing 25% of our total review.

### CPT1A P479L variant and unexpected infant death

CPT1A genotypes were available for 35 of the 81 unexpected infant deaths (SIDS/SUDI or infection) during the review period. Of the 35 cases with genotype, 30 were P479L homozygous and the remaining 5 were heterozygous. All SIDS/SUDI cases with CPT1A genotype data (17 P479L homozygous and 5 heterozygous) had other risk factors for sudden death present, including non-supine sleep position and/or bed-sharing at time of death. All infectious death cases were homozygous (8 respiratory and 5 *Haemophilus influenzae*).

P479L homozygosity was associated with unexpected infant death in Nunavut with all regions combined (OR: 3.43, 95%CI: 1.30-11.47; Table 2). Population based P479L homozygosity was significantly higher in the Kitikmeot and Kivalliq regions compared to the Qikiqtani region (χ^2^=22.86, p<0.0001), suggesting population stratification in the Qikiqtani region, where the variant is not in Hardy-Weinberg equilibrium [24].There was no statistical difference in homozygosity between the Kivalliq and Kitikmeot regions.

**Table 2:** Comparison of CPT1A P479L homozygosity in unexpected infant death cases (SIDS, SUDI and infection) occurring in Nunavut (July 1, 1999-June 30, 2011) to the estimated population P479L homozygosity [24], by region and territory

	**Cases**			**Population**			**Odds Ratio**	**(95%CI)**	**2 sided FET*p***
	**n**	***f***	**(95%CI)**	**n**	***f***	(**95%CI)**			
**NUNAVUT**	30	85.7	(69.7-95.2)	442	63.6	(59.9-67.2)*	3.43	(1.30-11.47)	0.006
Qikiqtani	8	88.9	(51.8-99.7)	162	53.6	(47.8-59.4)*	6.91	(0.90- 308.87)	0.044
Kivalliq	16	88.9	(62.5-98.6)	170	70.0	(63.8-75.7)	3.44	(0.77-31.45)	0.108
Kitikmeot	6	75.0	(34.9-96.8)	110	73.3	(65.5-80.2)	1.09	(0.19-11.47)	1.000
Kivalliq & Kitikmeot (combined)	22	84.6	(65.1-95.6)	290	71.2	(66.5-75.7)	2.22	(0.73-9.04)	0.178

* Genotype frequencies deviated from Hardy-Weinberg equilibrium (p<0.05) [24].

*f* = frequency. *CI* = confidence interval. *FET* = fisher’s exact test.

## Discussion

Indigenous populations worldwide experience infant and postneonatal mortality rates that are substantially higher than national averages [10,32-34]. This is true in the Inuit regions of Canada, including the Nunavut territory, where infant mortality rates are consistently 3 times the national rate [3,10]. Our review suggests an overall crude infant mortality rate slightly lower than reported previously, likely due to under ascertainment of neonatal deaths. However, postneonatal mortality rates were consistent with or higher than previous reports [3,10].

SIDS and deaths due to infections have been previously identified as the leading contributors to the higher rates of postneonatal mortality in indigenous populations of Western Australia and Alaska [33,34]. In this review, Nunavut rates of SIDS/SUDI (6.08/1,000) and infection (2.71/1,000) were significantly higher than national rates (2003–2007) and consistent with those previously reported for Inuit-inhabited regions of Canada (1990–2000) [10]. Additionally, the high rate of SIDS/SUDI in this review is consistent with or higher than those reported for indigenous populations in Western Australia (4.7/1,000; 1998–2001) and Alaska (3.6/1,000; 2000–2003) [33,34].

### Sleep environment in SIDS and SUDI cases

We report a SIDS/SUDI rate for Nunavut that is significantly higher than the national rate (OR=11.97, 95%CI: 8.92-15.79). Review of sleep environment at time of death showed 70% of SIDS/SUDI cases had two or more sleep-related risk factors present. Importantly, non-supine sleep position was present in 69% of cases with sleep-position documented, emphasizing the need for improved safe sleep messaging in Nunavut.

Bed-sharing was also prevalent in SIDS/SUDI cases (70% of those with bed-sharing information); however, a majority of cases bed-sharing at time of death had additional sleep-related risk factors present, including non-supine sleep position and/or bed-sharing with non-caregiver(s). This is consistent with the 2006 Aboriginal Children’s survey that reports caregivers of Inuit infants are less likely to place the infant supine for sleep when bed-sharing [16].

Although our data are limited, our results are consistent with other studies that suggest bed-sharing with additional risk factors present increases risk for SIDS/SUDI [18,19]. In cultures such as the Inuit culture, where mothers traditionally and continue to prefer to bed-share with infants, advising against bed-sharing under all circumstances may not be easily accepted. Health promotion strategies must consider cultural perspectives and a strong emphasis on risk reduction is crucial [35]. Better information regarding infant sleep practices in the overall population and at time of death is needed to assess risk effectively. In our review, many SIDS/SUDI case reports did not include full information on sleep environment at time of death.

### Infection

Infection was the second leading cause of infant mortality during the review period, and had a cause specific mortality rate of 2.71/1,000 live births. Respiratory infection comprised the majority of cases. Infants in Nunavut have the highest reported rate of hospitalisation for lower respiratory tract infections worldwide, with an average of 306-484/1,000 infants [36,37]. The hospitalization rate for infants with heart defects is even higher at 800/1,000 infants. Prematurity, tobacco smoke exposure (prenatal and postnatal), overcrowding and poor ventilation are all risk factors for respiratory infections and hospital admissions for Inuit children living in Nunavut [37,38]. Although tobacco smoke exposure data were limited in this review, environmental tobacco smoke is reported to be present in ~90% of Nunavut homes, which are small, overcrowded and have low air change rates [39].

*Haemophilus influenzae* infection (type a or b) was the cause of death for 6 cases during the review period. The International Circumpolar Surveillance system reports that northern Canada has the highest rate of invasive *Haemophilus influenzae* type A in children under the age of two in the circumpolar region (1999–2006) [40]. From 2009–2010, there were 11 cases under age 2 confirmed in Nunavut (G Osborne, personal communication).

### Prematurity

Nunavut has an average preterm birth rate of 12%, which is 1.5 times the Canadian average of 8% [31]. Infants born premature have a 3–6 times higher risk of mortality than term infants, including higher risks for deaths due to SIDS and infections [41,42]. Premature infants comprised at least 25% of the infant mortality cases in this review. This is likely an underestimate since the early neonatal deaths occurring out of territory were not available for review. High levels of prenatal exposure to cigarette smoke increases the risk of premature birth [41,43]. Although prenatal maternal smoking was not well documented in the data available for this review, in other studies 60-80% of Nunavut women self-reported smoking during pregnancy, almost 5 times the national average of 13% [43,44]. Mothers who reported smoking greater than 10 cigarettes per day during pregnancy had twice the risk of having a premature infant than those who did not smoke [43].

### P479L CPT1A homozygosity in unexpected infant death cases

CPT1A is an important liver enzyme required to transport long chain fatty acids into the mitochondrion for use as energy when dietary carbohydrates are unavailable (i.e. during fasting or prolonged exercise). CPT1A deficiency is a rare autosomal recessive disorder, usually presenting in infancy as non-ketotic hypoglycemia and metabolic decompensation triggered by fasting, which can progress to seizures, brain damage, and sudden death [45]. A small number of Nunavut Inuit infants and children, homozygous for the CPT1A P479L variant, have presented symptomatically with features consistent with CPT1A deficiency or with sudden unexpected death [30].

In our review, P479L homozygosity was associated with unexpected infant death (SIDS/SUDI and infection) in Nunavut as a whole (OR 3.43, 95%CI:1.30-11.47; p=0.006), which is consistent with results reported for Alaska Native and British Columbia First Nations populations [22,23]. However, analysis of the 2006 population data by region determined that the background prevalence of P479L homozygosity was significantly lower in the Qikiqtani region. Although underpowered, the association of P479L homozygosity with unexpected infant death was less apparent in the high prevalence regions, especially in the Kitikmeot region (OR: 1.09, 95% CI: 0.19-11.47), which had the highest rates of postneonatal mortality, SIDS/SUDI and infant death due to infection.

Although the overall population of Nunavut is small (~33,000) and the regions often combined, rates of immigration and emigration differ between the Nunavut regions [46]. Population stratification leading to spurious results is a particular concern in genetic association studies, especially when geographic sub-sets of small populations are being evaluated. The significantly lower P479L homozygosity in the Qikiqtani region may be due to admixture and/or a higher rate of non-Inuit immigration from other regions and provinces. Combining all regions increases the likelihood of a statistically significant difference if the case population rate is not entirely reflective of the reference population. Indeed, all postneonatal deaths in our review were born to Inuit mothers, which may have a higher homozygosity rate than the whole Qikiqtani region.

At this time we cannot rule out or confirm an associated risk with the P479L allele given our small numbers. Further study, controlling more precisely for population stratification is indicated. It remains possible that the association between the variant and unexpected infant death represents a complex interaction of the variant with environmental risk factors including sleep position, bed-sharing, prenatal and postnatal tobacco smoke exposure and food security. The ‘triple risk hypothesis’ for SIDS proposes that an external stressor combines with an underlying vulnerability at a critical development period to cause a SIDS death [47]. The P479L variant may represent an underlying vulnerability (i.e. a predisposition to hypoglycemia) that, when combined with exogenous stressors during a critical development period and/or intercurrent illness, may increase risk for unexpected infant death.

Alternatively, it is possible that the variant when homozygous serves as a marker of other social, economic, cultural or biological factors which increase the risk for infant mortality. There is suggestion of protective effect for cardiovascular lipid profiles and obesity in the Alaska Yupik and Greenland Inuit adults, perhaps in the context of a traditional diet [25,26]. Given the high frequency of the variant in Inuit populations pointing to an historical advantage, the current clinical significance of the variant remains unclear.

### Limitations

This was a retrospective case review with incomplete risk determinant information. Most mortality subgroups presented in this review were too small for complex statistical analysis. Full ascertainment for CPT1A P479L genotype, sleep position and bed-sharing was not possible. Ongoing study is needed to confirm these baseline results.

Our review was limited to those cases documented in Nunavut by the Chief Coroner’s Office and the Nunavut Bureau of Statistics, therefore our dataset under-represented deaths of cases born out of territory, especially with death during the first hospitalization (for example extreme prematurity and severe congenital anomalies). Therefore it is expected that the neonatal mortality rate is lower in our study than reported elsewhere [3,10].

Information on socioeconomic status (SES), including maternal education, household income and food security, were not available for this review. This may be an important limitation because of the known association with low socioeconomic status and infant mortality [48]. Of particular concern for this population are the results from the recent Inuit Health Survey showing that almost 70% of Nunavut households with preschoolers are either moderately or severely food insecure, which is more than 7 times the national average (9%) [49]. On-going research will address this limitation.

## Conclusion

Consistent with a previous study of Inuit birth outcomes in Canada [10], our study suggests a greater proportion of infants in Nunavut die of SIDS, SUDI or infection than infants in the rest of Canada. Factors such as sleep position and bed-sharing may play a role in these deaths. Although the P479L variant was associated with unexpected infant death in Nunavut as a whole, the association was less apparent when population stratification was considered. Population stratification as an underlying determinant of this association needs further assessment, as does the association of other potentially interactive risk factors. Prospective study of the health outcomes for P479L homozygous infants is planned to clarify the variant’s role in the health of these individuals, and the impact, if any, that treatment may have on these health outcomes.

Improved prenatal and postnatal data collection would enhance the understanding of the increased rates of infant mortality and inform prevention strategies. Efforts to improve maternal child health surveillance are now underway [50]. On-going assessment of the multiple risk factors involved in infant mortality will inform public health policy and prevention. Information regarding sleep position needs to be better communicated in a culturally appropriate manner. Messaging about infant care and sleep practices should come from within communities as well as from health care providers.

## Abbreviations

CPT1A: Carnitine palmitoyltransferase 1A; CI: Confidence interval; SIDS: Sudden infant death syndrome; SUDI: Sudden unexpected death in infancy.

## Competing interests

The authors declare that they have no competing interests.

## Authors’ contributions

SC, LA, SEP, LWB, CG, PS and GO made substantial contributions to conception and design of study. SC, PS, CG and GO participated in the acquisition of data. SC conducted statistical analysis and drafted and revised the manuscript. LA and GO participated in analysis and interpretation of data and revision of the manuscript. All authors read and approved the final manuscript.

## Pre-publication history

The pre-publication history for this paper can be accessed here:

http://www.biomedcentral.com/1471-2431/12/190/prepub
